# Changes in clinical depression following Sleeve Gastrectomy

**DOI:** 10.1002/edm2.282

**Published:** 2021-06-23

**Authors:** Milad Kheirvari, Taha Anbara

**Affiliations:** ^1^ Microbiology Research Center Pasteur Institute of Iran Tehran Iran; ^2^ Department of Surgery Erfan Niayesh Hospital Tehran Iran

**Keywords:** depression, obesity, sleeve gastrectomy

## Abstract

**Introduction:**

Bariatric surgery is safe and efficient surgical method for weight loss, but it is not free from complications. We aim to evaluate the prevalence of depression after Sleeve Gastrectomy (SG) in a narrow period of time on Iranian ethnicity.

**Methods:**

307 cases that underwent SG in Erfan Niayesh hospital were included. The questionnaire was based on International Classification of Diseases, Ninth Revision (ICD‐9) codes to define diagnoses. Screening follow‐up period was 20 to 24 months. The level of statistical significance was set at *p* < .05.

**Results:**

Of 307 subjects, preoperative depression was 30.2% and post‐operative depression was 37.7% (*p* = .025). Besides, BMI, dyslipidaemia, good feelings about body size and weight loss were statistically significant at *p* < .05.There was an increased risk of depression following the procedure mainly in divorced cases.

**Conclusion:**

The prevalence of clinical depression after sleeve gastrectomy was statistically significant and dependent on other variables. We provided guidance for people considering SG and their clinicians in terms of evaluating potential risks and benefits of surgery.

## Graphical abstract

The prevalence of clinical depression after surgery was statistically significant and dependent on other variables. We provided guidance for people considering SG and their clinicians in terms of evaluating potential risks and benefits of surgery.

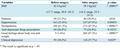

## 1 INTRODUCTION

The prevalence of obesity has tripled in the last 40 years,^1^ and almost half of the world's adult population is predicted to be overweight or obese by 2030.^2^ Obesity is not only a cosmetic concern, but also is a medical problem that increases the risk of other diseases, such as diabetes, cardiovascular diseases, mental problems and some kinds of cancers.^3^, ^4^, ^5^, ^6^ Based on the World Health Organization (WHO), obesity is defined as ‘abnormal or excessive fat accumulation that presents a risk to health’.^7^ Obesity results from a combination of inherited factors, environmental, socioeconomic, personal diet and exercise choice.^8^

Bariatric surgery is one of the safest and best choices for treatment of obesity and has evolved in the United States and worldwide over the past two decades and is done to help patients lose excess weight and reduce the risk of potentially life‐threatening weight‐related health problems. Bariatric surgery is a method of treatment that can be used in the case of patients with BMI ≥35 kg/m^2^ and with coexisting complications of obesity, such as arterial hypertension or diabetes, or with BMI ≥40 kg/m for normal individuals.^9^ However, the most popular bariatric surgery procedures are Roux‐en‐Y gastric bypass (RYGB) and sleeve gastrectomy (SG),^10^, ^11^, ^12^ these methods are not free from complications, and the most common ones are micronutrient deficiencies because of preoperative malnutrition, decreased food intake due to reduced hunger, and increased satiety and food intolerance or vomiting^13^ which lead to a series of mental problems such as depression after surgery.^14^, ^15^ Moreover, many patients have symptoms of depression after BS, but there are few prospective studies to specify their prevalence after surgery.^16^, ^17^, ^18^, ^19^ The neurological findings diagnosed with common post‐operative malnutrition include encephalopathy, polyradiculoneuropathy, optic neuropathy, myelopathy and polyneuropathy.^20^ These symptoms are more frequent in subjects who are noncompliant to medical visits in the long term after surgery.^12^ We aim to evaluate the prevalence of depression after SG in morbidly obese subjects in a narrow period of time on Iranian population.

## 2 MATERIALS AND METHODS

### 2.1 Data collection

In this retrospective study, data including enzymatic and hormonal parameters, anatomical imaging records and mental assessments were obtained from 307 adult individuals undergoing Bariatric surgery (Only SG) in Erfan Niayesh hospital between Oct 2017 and Jan 2020, according to the current procedural Terminology code: LSG (43,775). Approval for the use of the data in this study was obtained from the Erfan hospital, and all subjects signed patient consent forms to be involved in this survey.

### 2.2 Study subjects

All subjects were performed as a stand‐alone surgery and were evaluated by their surgeon and psychology team before and after the operation.

Obese subjects with BMI ≥ 35 kg/m^2^ and related comorbidities or BMI ≥ 40 kg/m^2^ with or without related comorbidities from 18 to 75 years of age were included in this survey. All information including age, BMI, gender, marital status, feelings about their weight and body size, anti‐depressant drugs prescription records, depression and any existing comorbidities including hypertension, diabetes mellitus and dyslipidaemia were recorded at the first preoperative appointment. Subjects with incomplete (<20 months of follow‐up) and unclear data or due exclusion criteria including any background of drug addiction, alcoholism, abnormal use of alcohol, hyperthyroidism and any psychological disorders rather than depression that made subjects unable to have stabilized mood were excluded. The follow‐up period defined at least 20 to 24 months. The term ‘Before surgery’ means 1–3 months earlier.

And the term ‘After surgery’ refers to 20–24 months post‐surgical follow‐up period.

### 2.3 Definition of depression

Data from preoperative psychological symptoms were collected in the bariatric clinic 1–3 months before undergoing the bariatric surgery. Based on previous reports, International Classification of Diseases, Ninth Revision (ICD‐9) codes were used to define diagnoses, while ICD‐9 and Current Procedural Terminology (CPT) codes were used to define procedures and interventions. Patients were asked to answer the questionnaires while waiting for the outpatient consultation by the physician or dietitian.

### 2.4 Study design

307 patients who underwent laparoscopic SG under supervision of Taha Anbara (M.D., General surgeon) and his team were considered as a study group in this survey. We included subjects who underwent SG in order to find out more psychological aspects of this method. We included Iranian population (Permanent citizens) and excluded other foreign individuals and populations in order to purify our study group. Also, we tried to include and distribute the statistical population somehow that we had equal classification of people from all three economic classes of low income, middle income and high income (Self‐reported).

### 2.5 Statistical analysis

All descriptive findings are presented as mean/median and SD for quantitative variables and count and percentage for qualitative variables. After checking the normality of variables using histogram graphs and the Kolmogorov‐Smirnov test, the Wilcoxon rank test was used to compare the non‐parametric variables and *t* test to compare other continuous variables before and after SG. The statistical significance level is defined as 0.05 (α = 0.05). Statistical analysis was performed using IBM SPSS Statistics 25 (SPSS Inc.).

## 3 RESULTS

Of 307 patients, 188 cases were females (61.2%) and 119 subjects were males (38.3%) and range from 21 to 75 years with 98 (31.9%) low income, 108 middle income (35.1%) and 101 (32.8%) high income subjects. Mean age was 51.47 years, and mean BMI was 42.4 kg/m2 (±7.7; range, 30.8–65.2). Preoperative characteristics for 307 subjects are illustrated in Table 1. The mean BMI after surgery was 29.3 kg/m^2^ (±4.3; range, 23.5–35.2) and statistically significant. Of 307 cases, there were 93 subjects (30.2%) that were diagnosed with symptoms of depression before surgery and 116 individuals (37.7%) suffering from depression after surgery in which the increase was statistically significant (*p* = .025).

**TABLE 1:** Characteristics of patients before surgery

Variables	*n*	%
Gender		
Female	188	61.2
Male	119	38.3
Marital status		
Married	172	56.0
Single	98	31.9
Divorced	37	12.0

We also evaluated the influence of BMI in relation to post‐operative clinical depression and significant differences were noted (*p* = .025). The resolution of diabetes and hypertension and reduction in anti‐depressant drugs prescription including sertraline (Zoloft), citalopram (Celexa), fluoxetine (Prozac) and paroxetine (Paxil, Pexeva) after surgery were not statistically significant. 189 subjects (61.5%) were diagnosed with dyslipidaemia before surgery, and this number decreased to 133 cases (43.3%) after surgery and this variable was significant in this population (*p* < .00001). On 307 cases, 268 subjects (87.2%) had good feelings about their weight and body size after surgery which was significant in statistical analysis, while just five participants (1.6%) stated good feelings about their bodies before surgery (*p* < .00001). More information on the prevalence of depression and other variables changes before and after SG is given in Table 2. There were no significant differences for the prevalence of depression and economic levels (*p *= 0.1247). Divorced subjects indicated more depression symptoms compared to other subjects (*p* .025).

**TABLE 2:** Comparison of depression and other variables changes before and after surgery among bariatric surgery subjects

Variables	Before surgery	After surgery	*p*‐Value
BMI	42.4 kg/m^2^ (±7.7; range, 30.8–65.2)	29.3 kg/m^2^ (±4.3; range, 23.5–35.2)	<.00001*
Diabetes	99 (32.2%)	86 (28.0%)	.0526
Hypertension	163 (53.0%)	147 (47.8%)	.09853
Dyslipidaemia	189 (61.5%)	133 (43.3%)	<.00001*
Anti‐depressant drugs prescription	64 (20.8%)	75 (24.4%)	.14231
Good feelings about body size and weight	5 (1.6%)	268 (87.2%)	<.00001*
Clinical depression	93 (30.2%)	116 (37.7%)	.025*

* The result is significant at *p* < .05.

## 4 DISCUSSION

Over the last two decades, the popularity of bariatric surgery, especially SG, has increased dramatically worldwide.^21^ Based on clinical studies, bariatric surgery is the most effective treatment for obesity.^13^, ^22^ The number of SG complications is relatively low, and with a rate of about 1.6% to 2.3%, this type of operation can be considered safe.^21^, ^23^ SG procedure is the choice of more than 70% of patients in the United States, Europe and Israel who tend to undergo obesity surgery which has been trending among various types of bariatric surgery in the past 10 to 15 years.^24^ Although bariatric surgery is safe and has many health benefits, as mentioned, there are some complications reported in patients, which nutritional deficiencies leading to clinical depression are one of the major complications in post‐operative periods.^13^ Depression after operation affects the quality of life and increases the cost and complications of surgery, among morbidly obese subjects,^25^ leading to enhanced rate of morbidity and mortality in surgery patients.^25^ Although depression symptoms cannot be completely prevented, finding the predictor factors and vulnerable population would be helpful for surgeons and psychologists to efficient management and prevention. We excluded patients with hyperthyroidism since hyperthyroidism and depression have overlapping features that can cause misdiagnosis.^26^ Obviously, this report is the first study of pre‐ and post‐operative psychological symptoms of SG in a long period of follow‐up time that mainly focuses on one type of surgery.

In this research, the study group was only Iranian ethnicity (Permanent citizens, people who were born and live in Iran) and excluded other foreign people in order to purify our study group. The main reason for the criteria was to avoid any interference in overall evaluation. For instance, people from other countries may be from different levels of economic situation, awareness and culture which may affect this analysis. Also, we tried to include and distribute the statistical population equally in three economic classes of low income, middle income and high income in order to evaluate the relationship of financial situation and clinical depression following sleeve gastrectomy. In this report, our main focus was on SG rather than other types of bariatric surgery. Therefore, our target group was the individuals who underwent SG in order to find out more psychological aspects of this specific method. We assumed the entire information from Jan 2020 would not be reliable due to the COVID‐19 pandemic and has been excluded from the study (increase in prevalence of depression due to quarantine, economic limitations and other situations among populace has been reported previously).

Although, there are several reports on bariatric surgery and psychological complications, but no comprehensive and specific study was found on the effect of SG on post‐operative depression on proper scale of subjects following SG. Based on a study on extremely obese patients who underwent gastric banding or gastric bypass, the prevalence of depressive disorders declined significantly after surgery.^27^ In another study, it has been claimed that bariatric surgery had a positive impact on depressive features among patient who underwent RYGB and laparoscopic adjustable gastric band (LAGB) procedures. However, data suggested some deterioration in improvement after the first post‐operative year in this survey.^28^ One another report stated that, RYGB, LAGB and SG surgeries, may lead to a mild alleviation in clinical depression in subjects over the initial post‐operative years but this was not maintained.^29^

In our study, comparing the pre‐ and post‐operative BMI, the results illustrate a significant weight loss from 42.4 kg/m^2^ before surgery to 29.3 kg/m^2^ after surgery (<.00001). As it has been mentioned, from 307 subjects, there were 116 subjects (37.7%) suffering from depression after surgery in this report. Albeit, the number of individuals undergoing gastric bypass or other types of bariatric surgery was high, and they were excluded from the study due to maintaining the heterogeneity of the population. In our retrospective analysis, the age factor, marital status, pre‐ and post‐operative BMI, anti‐depressant drugs prescription, good feelings about body size and weight and their impact on clinical depression were investigated in people who underwent SG procedure. The results were coincided with previously published studies on other methods.^30^, ^31^ However, most of the studies did not focus on depression and it was one of various psychological complications; in addition, they had a limited statistical population and follow‐up period.

However, due to the very low complication occurrence of SG and its numerous health benefits, patients and surgeons should focus more on complication prevention in vulnerable groups.

The strengths of our study were the higher number of patients of all ages, independent data collection, focus on specific type of bariatric surgery (SG), a defined surgeon, specific surgical team with the same surgical equipments for all patients, in an invariant hospital without interruption during the survey. As far as the weaknesses of this survey is concern, more than 60 months of follow‐up period, considering various aspects of psychological problems such as anxiety, eating disorders and memory loss can be taken into account in further studies.

## 5 CONCLUSION

This survey examined the prevalence of depression after SG in subjects with or without a prior history of depression. BMI, dyslipidaemia, good feelings about body size and weight and clinical depression changes were statistically significant at *p* < .05. There was an increased risk of depression following the procedure mainly in divorced cases. For patients considering bariatric surgery and their physicians, this research provides a clearer estimate of post‐surgical depression risk and associated exacerbating and mitigating factors. Other psychological impairments were not evaluated in this study.

## CONFLICTS OF INTEREST

There is no conflict of interests.

## AUTHORS CONTRIBUTIONS

TA designed and analysed the results, and MK performed and wrote the draft.

## ETHICAL APPROVAL AND CONSENT TO PARTICIPATE

The Ethics Committee (Helsinki board number: 2015073) approved this study. The protocol was approved by Tehran University of Medical Sciences Ethics Committee which is registered under the registration number IR.TUMS.VCR.PER.1399.123.

## CONSENT FOR PUBLICATION

Not applicable.

## Data Availability

Readers can reach the data and materials through direct contact to author's email.
